# An Investigation and Comparison of the Blending of LDPE and PP with Different Intrinsic Viscosities of PET

**DOI:** 10.3390/polym10020147

**Published:** 2018-02-05

**Authors:** Shi-Chang Chen, Li-Hao Zhang, Guo Zhang, Guo-Cai Zhong, Jian Li, Xian-Ming Zhang, Wen-Xing Chen

**Affiliations:** National Engineering Laboratory for Textile Fiber Materials and Processing Technology (Zhejiang), Zhejiang Sci-Tech University, Hangzhou 310018, China; scchenzstu@hotmail.com (S.-C.C.); zhanglihao0829@163.com (L.-H.Z.); 15858143135@163.com (G.Z.); zhongguocai1995@163.com (G.-C.Z.); 15857126685@163.com (J.L.); wxchen@zstu.edu.cn (W.-X.C.)

**Keywords:** polymer blend, poly(ethylene terephthalate), intrinsic viscosity, polyolefin, compatibilizer

## Abstract

The blending of aliphatic polyolefins and aromatic poly(ethylene terephthalate) (PET) based on different intrinsic viscosities (IV) was conducted in a torque rheometer. The comparison of blend components in terms of low density polythene (LDPE) and polypropylene (PP) in blending with PET was investigated, and the effects of the IV and proportion of PET on polymer blends are discussed in detail. Polymer blends with or without compatibilizer were examined by using a differential scanning calorimeter, thermogravimetric analyzer, rotary rheometer, field-emission scanning electron microscopy and a universal testing machine. It was found that the blending led to an increase in processability and a decrease in thermal stability for blends. The morphological analysis revealed that the incompatibility of blends was aggravated by a higher IV of PET, while this situation could be improved by the addition of compatibilizer. Results showed that there was an opposite effect for the tensile strength and the elongation at break of the polymer blend in the presence of a compatibilizer, wherein the influence of IV of PET was complicated.

## 1. Introduction

Polythene (PE) and polypropylene (PP), as representatives of aliphatic polyolefins, and poly(ethylene terephthalate) (PET), as a representative of aromatic polymers, are the three most common commercialized synthetic polymers, owing to their excellent overall performance and affordable price. The yield of the three polymers in China reached more than 14, 20, 40 million tons in 2016, respectively. PET can be categorized into three types with different intrinsic viscosities (IV) or molecular weights according to the application field: fiber grade, bottle grade and industrial yarn, whose IV are about 0.65 dL/g, 0.85 dL/g and 1.0 dL/g and whose corresponding aims are to be applied in the field of textiles, containers bottles and belts, respectively [1]. Occasionally, these three polymers can be used simultaneously in one product, for example, in beverage bottles. It also can be seen that recycled PET from different sources is basically a mixture of PETs with different properties [2].

The blending of polyolefins and PET can be an alternative to improve cost-effectiveness for industrial applications [3]. However, the thermodynamically immiscible nature of blend roots with large differences in their polarities, which leads to insufficient interfacial adhesion between blend components, result in poor mechanical properties [4,5,6]. Therefore, the polyolefin/PET compatibilization has already attracted a great deal of attention in the past decades.

Generally, the compatibilization of two immiscible polymers can be achieved by implementing a suitable grafting modification of polyolefin chains with reactive groups. The modification is achieved by chemical reactions between polar functional groups and polymer chains in melting blending [7]. By enhancing the interactions between grafted functional groups of polyolefin and PET end groups, the phase dispersion and adhesion at the interface can be improved [8]. For example, the functional groups like maleic anhydride (MAH) or glycidyl methacrylate (GMA) can be easily grafted onto polyolefins, and advantageously react with the carboxyl and hydroxyl end groups of PET. This method presents an excellent compatibilization effect with respect to chemical interactions between polyolefin and PET [9,10]. However, the conversion of functional groups grafted onto polyolefins is usually insufficient even though some residual active ingredients get involved in crosslinking reactions [11]. More importantly, it is not difficult to imagine that this route goes against the large-scale application due to its high cost. From this perspective, the grafted functional groups of polyolefins can be used as compatibilizers, with a small amount added into the PET/polyolefin blend during extrusion processes, then the desirable compatibilization effect can be obtained. The compatibilizer is concentrated at the interface of two phases in the blending system, and the co-crystallization/molecular chains’ entanglement between the polymer component of the compatibilizer and polymer matrix would be generated to facilitate the interfacial adhesion between two immiscible polymers. Consequently, two immiscible blending components are effectively compatible by forming a dispersed structure in one unity [12,13,14].

In the previous literature, GMA, MAH, acrylic acid (AA), ethylene-vinyl acetate copolymer (EVA), and maleimide (MI) are the common functional groups that can be either grafted onto polyolefins or copolymerized into compatibilizer. Recently, Jazani et al. [15] improved the mechanical properties of bottle grade PET by grafting GMA onto PP with the aid of styrene (St) comonomer, and found that the increase of impact strength was attributed to the enhanced interaction between epoxy groups of GMA and carboxyl end groups of PET, as well as the hydrogen bonds formed between PP-*g*-GMA and PET. In yet another approach, a compatibilizer composed of styrene–ethylene–butylene–styrene (SEBS) grafted with MAH was used as the compatibilizer in the blending of PP and PET spun fibers [16]. Results showed that the compatibilizer significantly affected the elongation at break of the blend, while no relevant effect could be observed in the anisotropically-oriented fibers. Using PP-*g*-AA as a compatibilizer, a good interaction between the PP and PET surfaces was achieved as PET islands was deformed in micro-fibers after hot stretching [17]. 

In most published works, PP is the dominant continuous phase in the blend system of polyolefins/PET. There is little research focusing on PE/PET blends, let alone on the compatibilizer, by grafting the functional groups onto PE. Lei et al. [18] studied the compatibility between PET and HDPE using PE-*g*-MAH, SEBS-*g*-MAH and E–GMA, respectively. It was found that the compatibilizing effectiveness of E–GMA was the best. Raffa et al. [19] indicated the chemical reactions among polymer and additives had a significant effect on the ultimate melt rheology and mechanical properties of recycled PET/polyolefin blends.

In most cases, a variety of nanomaterials as a third component are added into two immiscible polymers to improve the compatibilizing effect [20]. As a matter of fact, the functional groups grafted onto polyolefins have been commercialized and applied gradually, such as PP-*g*-MAH and PE-*g*-MAH, which offer the best convenience for designing polymer blend systems.

Based on the successful surface functionalization of polypropylene/polystyrene and their application in polymer blends, as described in our previous work [7,21,22,23,24], the motivation has turned to developing possible industrialization polymer blends by exploring the relationships between the structure and properties in the processable polymers. Consequently, this work concentrates on the comparative investigation of the blending of PP and LDPE with different IV of PET using commercialized polyolefin-*g*-MAH as a compatibilizer. A systematic performance investigation of the polymer blends with or without compatibilizer was performed to elucidate the processable correlation between several major polymer commodities. 

## 2. Materials and Methods

### 2.1. Materials

Three poly(ethylene terephthalate) (PET) chips with different intrinsic viscosities (IV) of 0.7 dL/g, 0.85 dL/g and 1.0 dL/g, were purchased from Zhejiang Guxiandao Polyester Dope Dyed Yarn Co., Ltd. (Shaoxing, China). The moisture and carboxyl end group content of the PET chips are also listed as Table 1. LDPE (F401) and PP (DNDB7441) were purchased from Sinopec Yangzi Petrochemical Co., Ltd. (Nanjing, China), LDPE-*g*-MAH and PP-*g*-MAH were purchased from Fine-blend Compatibilizer Jiangsu Co., Ltd. (Nantong, China). 

In order to decrease the hydrolytic decomposition, the PET chips were dried at 130 °C for 9 h in a vacuum oven before being blended into either LDPE or PP. The polymer blends were processed in a RM-200C torque rheometer (Hapro, Haerbin, China). The processing temperatures were set to 260 °C for all blends in the rheometer, and the processing time was set to 5~10 min and the rotor speed was set to 50 r/min. The total weight of polymer blend was no more than 50 g. The weight ratio of the components in the blending are listed in Table 2. The compatibilizer usually accounts for five percent of the total weight of the two polymers (5 wt %). The blank control experiments were also carried out in the absence of compatibilizer.

### 2.2. Measurements

The thermal behavior of the blends was investigated using differential scanning calorimeter (DSC1, Mettler Toledo, Schwerzenbach, Switzerland) and thermogravimetric analyzer (TGA, Mettler Toledo, Schwerzenbach, Switzerland) under nitrogen atmosphere. The drying samples were initially heated from 25 to 300 °C at a rate of 50 °C/min. After that, the samples were kept at the final temperature for 3 min to eliminate the thermal history, and then cooled to 50 °C at a rate of 20 °C/min to determine the crystallization temperature (*T_c_*) and before being kept for 3 min. Finally, the samples were heated to 300 °C at a rate of 10 °C/min to determine the melt point (*T_m_*). About 5 mg of the samples was heated from 25 to 600 °C at a rate of 10 °C/min with a nitrogen flow rate of 45 mL/min to determine the decomposition temperature (thermal gravimetric rate 5%, *T*_5%_).

The rheological properties of the polymer blends as functions of angular frequency were investigated via plate-plate geometry (plate radius = 25 mm; gap = 1 mm) using a stress-controlled rheometer (MCR 301, Anton Paar, Graz, Austria) at 260 °C. The measurements of the granules and sheet samples were performed in air atmospheres. The loaded frequency sweep was undirectional from the angular frequency of ω = 500 1/s to 0.1 1/s.

The banded samples of polymers were prepared from a blending system so that the morphological characteristics of the polymer blends could be examined using an ULTRATM 55 field-emission scanning electron microscopy (SEM Ultra 55, Zeiss, Jena, Germany) at an acceleration voltage of 1 kV. The samples were cooled in liquid nitrogen for 15 min and then taken out quickly to be brittle fractured. The fracture surface of PET was etched by using the mixed solution of phenol and tetrachloroethane (1/1 (wt %)), then the fracture surface was gilded because of the poor electrical conductivity.

To test the mechanical property of the polymer blends, the round rod samples with diameter of 1.5 mm were stretched on a universal testing machine (Instron 3367, Instron, Canton, USA) at speed of 10 mm/min. Generally, the effective stretch length was about 2 mm, and then the tensile strength and elongation at break could be achieved.

## 3. Results and Discussion

### 3.1. Thermal Properties

**Figure 1:**
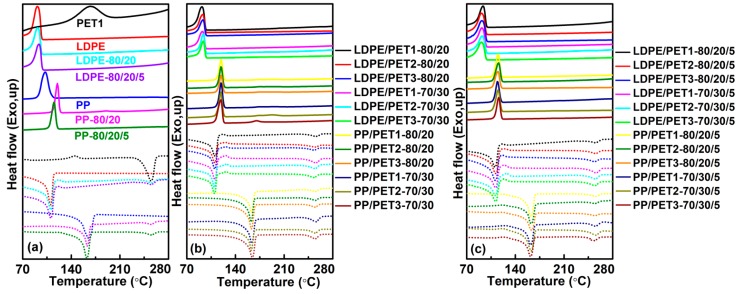
Cooling (solid line) and heating (short dot line) DSC scans of polymers and polymer blends, (**a**) polymers and blends; (**b**) incompatible polyolefin/PET blends; (**c**) compatibilized polyolefin/PET blends.

Figure 1 shows differential scanning calorimeter (DSC) scans of polymers and polyolefin/PET blends. The crystallization could be observed in the cooling scan and the melting process was recorded in the subsequent heating scans. The evident cold crystallization peaks (*T_c_*_1_) of the polymer blends, observed in Figure 1a, was between polyolefins and PET, while the second cold crystallization peaks (*T_c_*_2_) was almost invisible in most cases. In contrast, there were two melting peaks in the heating DSC scans, which indicated the melting point of the polyolefin component (*T_m_*_1_) and PET component (*T_m_*_2_). Noticeably, the *T_c_*_1_, *T_m_*_1_ and *T_m_*_2_ for all polyolefin/PET blends were listed in Table 3. It also could be seen that blending caused a gradual increase in the crystallization temperature (*T_c_*_1_) from LDPE to LDPE/PET1, to LDPE/LDPET-*g*-MAH/PET, however, the *T_c_*_1_ of compatibilized PP/PET1 blends was lower than that of incompatible PP/PET1 blends. This might be ascribed to the plasticizing effect of compatibilizer on the PP/PET blend and the facilitation of crystallization [25]. On the other hand, it is well known that the regularity of the LDPE chain is better than PP, which increases the crystallization temperature of blending system.

Generally, the *T_c_*_1_ of incompatible LDPE/PET (Figure 1b) increased slightly, yet the *T_m_*_1_ and *T_m_*_2_ presented a gradual decline either with the increase of the content of PET or with the increase of the IV of the PET, but there was less significant difference among PP/PET blends. When the compatibilizer was added to two immiscible polymers (Figure 1c), the *T_c_*_1_ of LDPE/PET blends presented a minor depression, whereas the changing trends for the PP/PET blends is just opposite. It is worth stressing that the difference in the *T_c_*_1_, *T_m_*_1_ and *T_m_*_2_ of the polymer blends was quite inconspicuous, and that the thermal behaviors need further verification. This is perhaps explained by the fact that both the content and property of PET have no significant influence on the cold crystallization performance of polyolefins/PET. These allow us to conduct a good deal of the blending of polyolefin with PET from different sources. 

**Figure 2:**
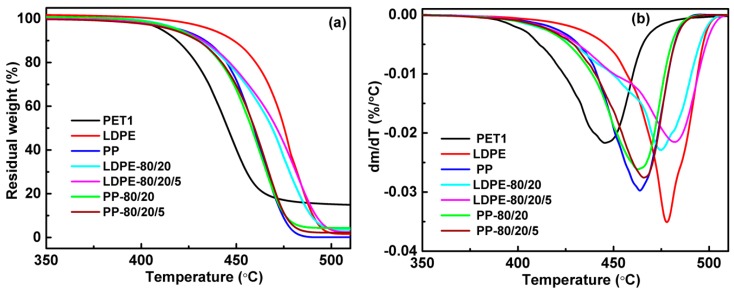
Comparison of TG (**a**) and DTG (**b**) curves of polymers and polymer blends.

The comparison of thermogravimetric (TG) and derivative thermogravimetric (DTG) scans of polymer with polymer blends is reported in Figure 2. PET presented decomposition at lower temperatures than LDPE and PP, as a result of the relatively poor stability of the ester group in PET chains. A small difference in the decomposition rate could be seen between PP and its blends based on the TG and DTG curves. Nevertheless, it was clear that the LDPE/PET blend showed worse thermal stability than that of LDPE and that the compatibilizers promote the thermal stability of two immiscible polymers. This was mainly attributed to the improvement of compatibility with the addition of grafting maleic anhydride; the interface adhesion was enhanced by the chemical reactions between the grafted functional groups of polyolefin and PET end groups [26].

**Figure 3:**
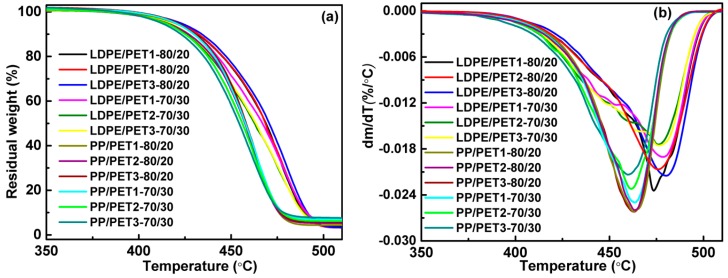
TG (**a**) and DTG (**b**) curves of incompatible polymer blends.

**Figure 4:**
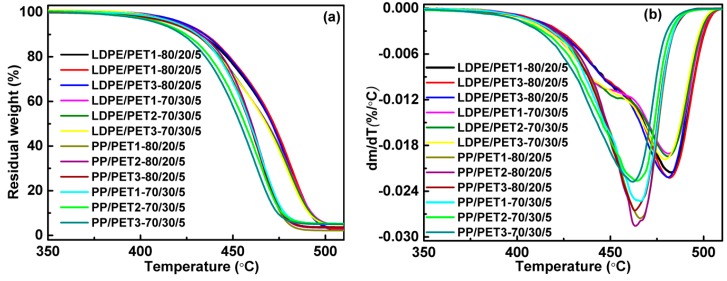
TG (**a**) and DTG (**b**) curves of compatibilized polymer blends.

Figure 3 and Figure 4 show the effect of the content and IV of PET on the thermal stability of incompatible and compatibilized polyolefin/PET blends, respectively. The DTG curves of polymer blends presented asymmetric peaks, which indicated the whole decomposition of the different components. Accordingly, the decomposition temperature at thermal gravimetric rate of 5% (*T*_5%_) and the corresponding temperature of the maximum decomposition rate (*T_cmax_*) of all polymer blends are also listed in Table 4. Regarding LDPE/PET blends without a compatibilizer (Figure 3), the decomposition temperature was slightly increased with the increase in the IV of PET, which was validated by the temperature of maximum decomposition rate as shown in the DTG graphs and Table 4, but the difference was unremarkable under the conditions of more PET content in the blends. On the contrary, it almost stayed at a constant thermal gravimetric rate for PP/PET blends in a ratio of 80 to 20 as the IV of PET increased, while it was a little better later with more PET content in the blends. When the compatibilizer was added to the blends (Figure 4), the thermal stability was slightly increased. As already mentioned, the interface could be enhanced. It was also noticed that both the maximum decomposition rate and the corresponding temperature of the compatibilized polyolefin/PET blends at a ratio of 70 to 30 were lower than that of 80/20 PP/PET blends in view of the poorer thermal stability of PET. Furthermore, the polymer blends with a higher IV of PET generally suggested better thermal stability and higher value of *T*_5%_ and *T_cmax_*. These results are easy to understand if it is remembered that PET with a higher IV has a longer polymer chain. 

### 3.2. Analysis of Rheological Behavior

**Figure 5:**
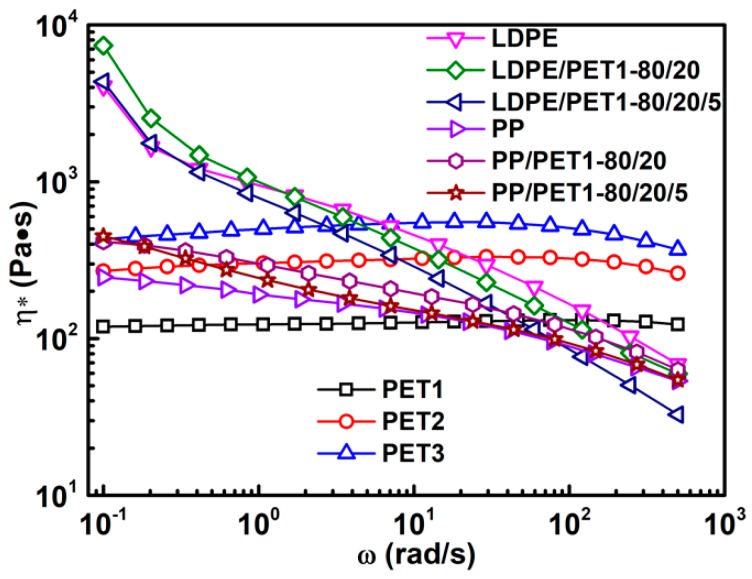
Complex viscosity for polymers and polymer blends.

Figure 5 shows the complex viscosity as a function of frequency at 260 °C for polymers and polymer blends. The rheological curves of polymer blends showed similar trends with the neat constituents of polymers. Considering that the *T_m_* of PET was approximately equal to 260 °C, all the rheological measurements of polymers and polymer blends were conducted in air without inert gas protection, which was consistent with the blending process. Three kinds of PET displayed a pseudo-Newtonian behavior for the whole frequency range. The complex viscosity significantly increased with the IV of PET due to the longer polymer chain [1]. Both the polyolefins and polymer blends presented remarkable pseudoplastic behavior, and LDPE and its blends revealed a stronger shear thinning effect than PP and its blends because of the better chain mobility.

Compared with LDPE, the incompatible LDPE/PET blend showed a higher viscosity at low frequency and a lower viscosity at high frequency. However, the complex viscosity of compatibilized LDPE/PET blend was always lower than that of LDPE except at an extremely low frequency, and also lower than that of incompatible LDPE/PET blend within the whole frequency range. However, once the compatibilizer was formally incorporated into the system, the viscosity of the system decreased, which was due to the role of the compatibilizer in reducing the dispersity and inducing interaction at the interface between blend components, eventually increasing the mobility of the compatibilized system [27,28]. PP showed the lowest viscosity at low frequency, and the compatibilized PP/PET blend presented intermediate behavior between the incompatible blend and pure PP. As mentioned above, the carbonyl group in PET could interact with the pendant group in the PP chain so that the blending system gets less viscous, whereas the compatibilizer played a role of softening agent [29].

**Figure 6:**
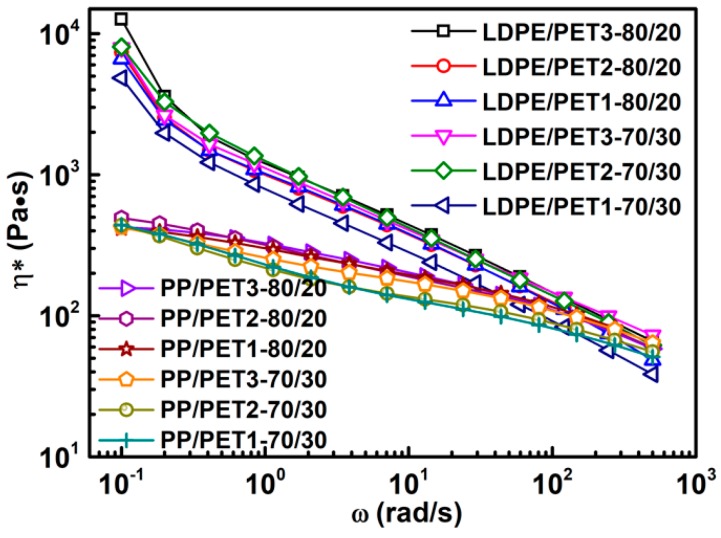
Complex viscosity for polymer blends with different IV of PET without compatibilizer.

Figure 6 shows the complex viscosity of incompatible polymer blends. It can be seen that the viscosity of both the LDPE/PET and PP/PET blends present a slight increase as the IV of PET increases. This is probably due to the fact that the higher IV of PET has a longer polymer chain so that the PET itself possesses a higher viscosity. However, the blends with a higher content of PET did not exhibit a higher viscosity but displayed a slight decline. This suggested that the PET dispersed in polyolefins had some lubrication effect in a way due to the presence of flexible chain segments in PET.

**Figure 7:**
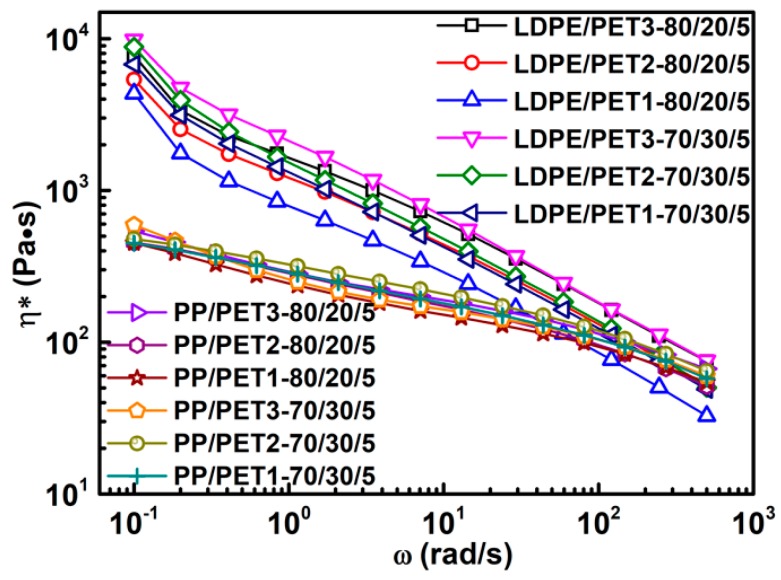
Complex viscosity for polymer blends with different IV of PET in the presence of compatibilizer.

Figure 7 shows the complex viscosity for polymer blends with different IV of PET in the presence of compatibilizer. It was in good agreement with the viscosity sequence of incompatible polymer blends with different IV of PET. Moreover, it was noticed that the viscosity of blends with a higher content of PET was significant increased. Several studies have shown evident results that polyolefin/PET blends are completely immiscible. By adding compatibilizer into the blending system, the interaction between the compatibilizer and blend components led to a reduction of interfacial tension and the suppression of coalescence [3,30,31,32]. Hence, it could be inferred that with the increase of PET content, the compatibilizer provided increasingly favorable support for an enhanced interaction between blend components.

### 3.3. Morphological Characterization of Polymer Blends

**Figure 8:**
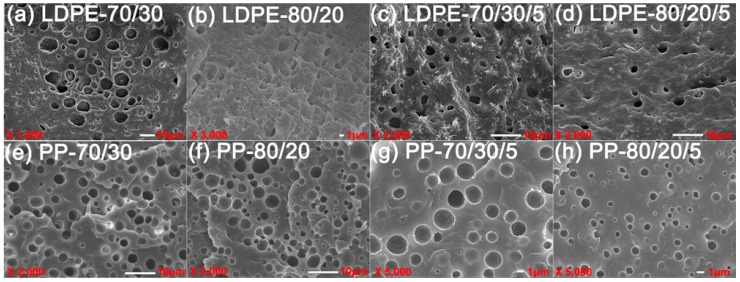
SEM micrographs of incompatible (**a**,**b**,**e**,**f**) and compatibilized (**c**,**d**,**g**,**h**) polymer blends.

Figure 8 shows the morphology of incompatible and compatibilized polyolefin/PET1 polymer blends. It can be seen that the blending of two immiscible components results in an entirely clear phase interface. The cross profile of LDPE/PET was a brittle fracture and PET dispersed unevenly in LDPE phase in the form of particles. In yet another way, the large size of the circular hole indicated that the PET-dispersing phase appeared in the PP continuous phase, resulting in a typical sea-island structure. Figure 8 also shows the scanning electron microscopy (SEM) micrography of different amounts of PET with or without compatibilizer. With the increase of PET content, the holes of the LDPE/PET and PP/PET blends were increased in quantity, size and dispersion uniformity. The blends with a higher content of PET were more likely to be assembled in the blending process, resulting in the enlargement of the dispersed phase size. Compared to incompatible polymer blends, it was observed that compatibilized LDPE/PET blends had a better dispersity of holes appearing in the cross profile, the fracture surface was no longer clear but presented a significant layered hippocampal structure, and the circular hole in LDPE/PET blends with PP-*g*-MAH tended to be dispersed uniformly with diminishing size. All these confirmed that the compatibilizer had indeed improved the compatibility between polyolefins and PET. As mentioned above in the analysis of rheological behavior, the compatibilizer enhanced the interaction between the blend components which decreased the interfacial tension and suppressed the coalescence [3,30,31,32].

**Figure 9:**
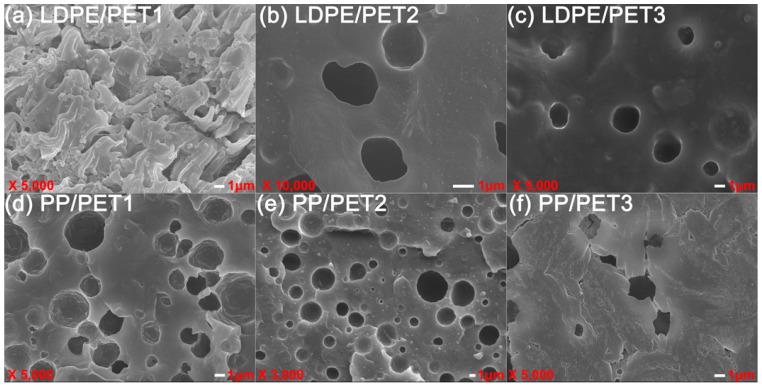
SEM micrographs of LDPE/PET (**a**–**c**) and PP/PET (**d**–**f**) blends with different IV of PET without compatibilizer (80/20).

**Figure 10:**
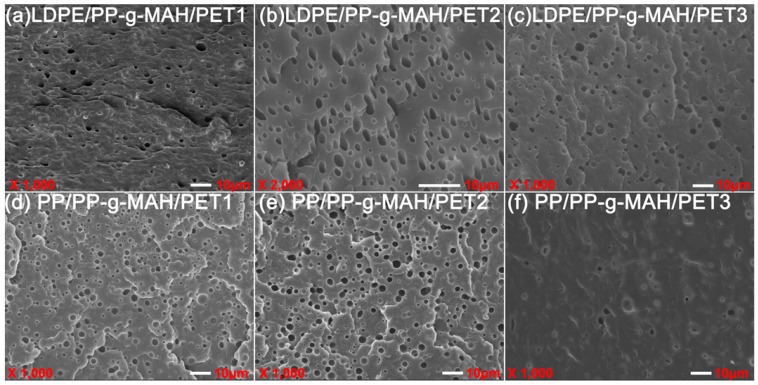
SEM micrographs of LDPE/PET (**a**–**c**) and PP/PET (**d**–**f**) blends with different IV of PET in the presence of compatibilizer (80/20/5).

The effect of the addition of different IV of PET on the blending system with or without compatibilizer was visualized by morphological characterization, as shown in Figure 9 and Figure 10, respectively. In Figure 9, the dispersed phase in PP/PET blends was getting more and more clear with the increase in the IV of PET; by contrast, the phase separation of the two blend components was attenuated gradually, so that a rough interface and more crack propagation could be seen in PP/PET blends. This could be explained in that it was more difficult to diffuse into the polyolefin phase for a longer polymer chain of PET, as a consequence, the thinner interface layer suggested poor compatibility. With the addition of compatibilizer (Figure 10), the PET dispersed uniformly and the interface coalescence was strengthened. Not surprisingly, in this situation, the effect of the IV of PET on the dispersed phase size was weakened.

### 3.4. Mechanical Properties of Polymers before and after Blending

Table 5 and Table 6 show the elongation at break and the tensile strength of different polymer blends, respectively. The pure PET exhibited a decrease of elongation at break and an increase of tensile strength with the increase of IV. The polymer blends with a higher content of PET had a relatively lower elongation at break. As an immiscible polyolefin/PET blend, the interphase adhesiveness of two blending components was insufficient, which resulted in an evident interphase division, and therefore, worse interphase adhesiveness existed in polymer blends with higher content of PET. 

Without compatibilizer, the elongation at break of both LDPE/PET blends and PP/PET blends declined with the increase of the IV of PET. Yet it increased remarkably after the compatibilizer was added to polyolefin/PET blends, especially for the highest IV of PET3 in blends. With longer polymer chains and higher steric effects, the PET with a higher enlarged interphase between the polyolefins and the PET, showed a decrease of elongation at break. The compatibilized polymer blends were expected to have a higher elongation at break because of the increased interphase adhesiveness and enhanced interaction. In this case, PET3 with better mechanical properties had a positive effect on the elongation at break of blends. This is different from the PE/PET blend with organoclay as a compatibilizer, as there may be a possible degradation of the clay surfactant during melt compounding [33]. It was noted that the elongation at break of the LDPE blending system was higher than that of the PP blending system, which was consistent with the observation of the rheological behavior which found that LDPE/PET blends had the benefit of higher viscosity. In addition, the elongation at break of the polyolefin/PET blend increased with the increase of PET dispersed phase content, which was in good agreement with the results of the research by Tavanaie et al. [34].

The variation of the tensile strength of polymer blends with PET content was largely different from the elongation at break, in that it was somewhat confused. In such a situation, the polymer blends were brittle fractured to subdue the tenacity of the materials. With the increase of the IV of PET, the tensile strength of the LDPE/PET blends was decreased, while it was gradually increased for PP/PET blends. According to the SEM micrographs, the interphase adhesiveness of incompatible LDPE/PET blends was relatively weaker than that of PP/PET blends. However, with the addition of compatibilizer, LDPE/PET blends showed better tensile properties since the shorter chain segment of LDPE-*g*-MAH was more likely to diffuse successfully into two blend components. It was also noticed that the tensile strength of all polymer blends generally declined, and the difference of tensile strength caused by either changing the IV of PET or changing the PET content also decreased. Therefore, it can be stated that the compatibilizer played a role in increasing interphase adhesiveness to prevent brittle fracture, thus increasing the mechanical properties of polymer blends.

## 4. Conclusions

The blending of LDPE and PP with different intrinsic viscosities (IV) of PET was performed in the absence or presence of compatibilizer. The impact of the blending components on the thermal, rheological, morphological and mechanical properties was discussed in detail, and the following conclusions were drawn from the discussion: 

(1) The blending leads to a higher crystallization temperature of the polyolefin component, yet the melting peak both of the polyolefin component and of the PET component in the blends are little different from their pure polymer. While the thermal stability of polymer blends is generally better than PET but worse than LDPE, and blending of PP with PET had no significant effect on the thermal stability of PP. The thermal performance of polyolefin/PET was slightly decreased by either the increase of the IV of PET or the decrease of PET content. It was found that the compatibilizer can improve the thermal stability of the blending system by enhancing the interface adhesion. 

(2) The complex viscosity of incompatible polymer blends is slightly higher than that of compatibilized blends. The results show that it is increased with the increase of the IV of PET, yet decreased with the increase of PET content increasing in blends. The complex viscosity of the blend with compatibilizer can be similarly increased by either increasing the IV of PET or increasing the PET content in blend. 

(3) PET disperses nonuniformly in LDPE in the form of granules; the cross-section of the incompatible blend shows brittle fracture. The number of holes in the blend increases with the increase of PET content. In contrast, the cross-section tends to be indistinct in the presence of LDPE-*g*-MAH as a compatibilizer. As the IV of the PET increases in the polymer blends, PET can be easily gathered to enlarge the scale of the dispersed phase, resulting in worse nonuniform dispersity, while it can be improved by adding compatibilizer.

(4) It was demonstrated that with the increase of the IV of PET, the elongation at break of the polymer blend decreases slightly and the tensile strength of the LDPE/PET blend also decreased accordingly, while the tensile strength of the PP/PET blend gradually increased. It is worth noting that the higher content of PET in the blend results in a smaller elongation at break and lower tensile strength. When the compatibilizer was added to the blends, an increase of elongation at break and a decrease of tensile strength could be seen simultaneously, and the difference of tensile strength caused by either changing the IV of PET or changing the PET content declined.

## Figures and Tables

**Table 1:** The properties of the PET chips.

PET	[η] (dL/g)	[COOH] (mol/t)	H_2_O (wt %)
PET1	0.70	22	0.35
PET2	0.85	18	0.26
PET3	1.00	15	0.10

**Table 2:** The weight ratio of the components in the polymer blends.

No.	PET1	PET2	PET3	PP	PP-*g*-MAH	LDPE	LDPE-*g*-MAH
1	20			80			
2		20		80			
3			20	80			
4	30			70			
5		30		70			
6			30	70			
7	20			80	5		
8		20		80	5		
9			20	80	5		
10	30			70	5		
11		30		70	5		
12			30	70	5		
13	20					80	
14		20				80	
15			20			80	
16	30					70	
17		30				70	
18			30			70	
19	20					80	5
20		20				80	5
21			20			80	5
22	30					70	5
23		30				70	5
24			30			70	5

**Table 3:** The crystallization temperature and melting point of polymer blends.

Samples	*T_c_*_1_ (°C)	*T_m_*_1_ (°C)	*T_m_*_2_ (°C)
LDPE	91.09	110.12	—
PP	102.51	165.33	—
PET1	167.69	—	254.33
LDPE/PET1-80/20	91.85	110.68	255.67
LDPE/PET2-80/20	92.96	110.58	254.81
LDPE/PET3-80/20	93.43	110.23	253.32
LDPE/PET1-70/30	92.49	110.77	2557.5
LDPE/PET2-70/30	93.44	109.68	254.34
LDPE/PET3-70/30	94.44	109.86	252.88
PP/PET1-80/20	119.61	163.31	255.28
PP/PET2-80/20	119.14	162.96	255.25
PP/PET3-80/20	119.21	163.33	254.87
PP/PET1-70/30	119.13	163.70	255.75
PP/PET2-70/30	118.67	162.96	254.83
PP/PET3-70/30	118.65	164.42	254.81
LDPE/PET1-80/20/5	93.61	110.63	255.18
LDPE/PET2-80/20/5	91.39	111.66	255.96
LDPE/PET3-80/20/5	92.13	111.13	253.88
LDPE/PET1-70/30/5	91.78	111.40	256.22
LDPE/PET2-70/30/5	92.32	111.63	254.93
LDPE/PET3-70/30/5	91.23	111.66	254.66
PP/PET1-80/20/5	114.91	162.62	255.96
PP/PET2-80/20/5	115.61	162.86	254.93
PP/PET3-80/20/5	115.07	163.39	254.66
PP/PET1-70/30/5	114.36	162.09	255.44
PP/PET2-70/30/5	115.45	162.87	255.71
PP/PET3-70/30/5	116.00	162.09	253.88

**Table 4:** The decomposition temperature (*T*_5%_) and the corresponding temperature of the maximum decomposition rate (*T_cmax_*) of polymer blends.

Blend	0/100		80/20		70/30		80/20/5		70/30/5		100/0	
	*T*_5%_ (°C)	*T_cmax_* (°C)	*T*_5%_ (°C)	*T_cmax_* (°C)	*T*_5%_ (°C)	*T_cmax_* (°C)	*T*_5%_ (°C)	*T_cmax_* (°C)	*T*_5%_ (°C)	*T_cmax_* (°C)	*T*_5%_ (°C)	*T_cmax_* (°C)
LDPE/PET1	410.8	444.3	419.7	473.4	420.5	478.7	419.6	482.8	419.4	481.7	437.5	477.7
LDPE/PET2	407.5	445.7	421.3	475.9	416.3	476.0	423.2	481.7	420.3	480.9	-	-
LDPE/PET3	407.2	447.7	422.5	480.3	416.4	475.8	423.6	480.8	419.5	479.2	-	-
PP/PET1	410.8	444.3	419.5	463.5	420.3	463.3	416.3	465.9	419.7	465.7	420.8	461.3
PP/PET2	407.5	445.7	417.1	463.4	414.3	461.4	421.1	463.3	411.5	465.0	-	-
PP/PET3	407.2	447.7	420.7	463.2	412.1	460.5	417.5	462.9	409.9	461.8	-	-

**Table 5:** The elongation at break of polymer blends.

Blend	0/100 (%)	80/20 (%)	70/30 (%)	80/20/5 (%)	70/30/5 (%)	100/0 (%)
LDPE/PET1	8.55	51.13	25.51	67.45	37.52	223.45
LDPE/PET2	8.45	44.67	23.50	78.25	41.47	-
LDPE/PET3	6.41	44.16	22.21	148.54	58.97	-
PP/PET1	8.55	15.1	11.7	38.5	30.5	428
PP/PET2	8.45	14.7	11.5	34.8	28.0	-
PP/PET3	6.41	13.7	10.2	31.3	24.3	-

**Table 6:** The tensile strength of polymer blends.

Blend	0/100 MPa	80/20 MPa	70/30 MPa	80/20/5 MPa	70/30/5 MPa	100/0 MPa
LDPE/PET1	26.0	16.71	23.52	14.32	18.25	12.31
LDPE/PET2	28.8	15.62	17.25	13.88	12.74	-
LDPE/PET3	37.5	14.45	17.01	12.90	12.24	-
PP/PET1	26.0	17.1	24.2	11.3	12.5	35.1
PP/PET2	28.8	20.6	25.0	11.9	12.41	-
PP/PET3	37.5	24.4	25.2	12.0	12.2	-
